# The prevalence of neurodevelopmental disorders in Smith-Magenis Syndrome: a PRISMA compliant systematic review

**DOI:** 10.1186/s11689-026-09707-y

**Published:** 2026-06-01

**Authors:** Pauline Boiroux, Marie-Noëlle Babinet, Gabrielle Chesnoy, Paul Belhouchat, Caroline Demily

**Affiliations:** 1 Centre de Référence Maladies Rares Troubles du Comportement d’Origine Génétique (GénoPsy), Le Vinatier Psychiatrie Universitaire Lyon Métropole, 95 Boulevard Pinel, Bron, 69500 France; 2 Institut des Sciences Cognitives Marc Jeannerod, UMR 5229, CNRS & Université Claude Bernard Lyon 1, Université de Lyon, 67 Boulevard Pinel, Bron Cedex, 69675 France; 3 Centre d’information et de documentation - CRA Rhône-Alpes - Le Vinatier Psychiatrie Universitaire Lyon Métropole, Bât. 211 - 95, boulevard Pinel, Bron Cedex, 69677 France

**Keywords:** Smith Magenis Syndrome, Autism Spectrum Disorder, Attention Deficit Hyperactivity Disorder, Intellectual Developmental Disorder, Neurodevelopmental Disorders, Prevalence, Systematic Review

## Abstract

**Background:**

Smith Magenis Syndrome is either due to a deletion in 17p11.2 locus or to a pathogenic variant in *RAI1* gene and is associated with a higher risk of neurodevelopmental disorder. We performed a systematic review to assess the prevalence of neurodevelopmental disorders (autism spectrum disorder, attention deficit hyperactivity disorder, intellectual developmental disorder, specific learning disabilities) in Smith Magenis Syndrome as a main outcome. We described the methods used to identify a neurodevelopmental disorder in this population and assessed a phenotype-genotype correlation as secondary outcomes.

**Methods & results:**

Main electronic databases were searched on January 31^st^, 2024. We considered original articles published in peer-reviewed journals. We selected studies with data concerning the prevalence of neurodevelopmental disorders in people with Smith-Magenis Syndrome. Quality was assessed based on sample identification, confirmation of syndrome, assessment of neurodevelopmental disorders and data coverage. 1941 articles were identified and 14 were included in this review. Data were synthesized and displayed as descriptive tables. No meta-analysis was performed due to the paucity of studies. In total, 451 patients presenting with Smith Magenis syndrome were assessed for neurodevelopmental disorders. 6 studies (*n*=220 patients) assessed autism spectrum disorder characteristics with prevalence ranging from 14% to 95%, and mean prevalence of 43% and 80% for deletion and *RAI1* samples respectively. 6 studies assessed attention deficit hyperactivity disorder characteristics (*n*= 174 patients). Only one study assessed ADHD using standardized clinical criteria, reporting 100% prevalence. Other studies assessed impulsivity, hyperactivity, attention deficit with mean proportions of 78%, 76% and 96% respectively. 9 studies reported IDD prevalence, ranging from 67% to 100%. For deletion carriers, pooled prevalence was 93%, mostly moderate (43%), while 80% was reported for *RAI1* carriers, mostly mild (69%). Specific learning disabilities were described for 9% of deletion 17p11.2 patients. Methods of assessment were very heterogeneous, consisting in the main limit of this study along with the paucity of articles included.

**Conclusions:**

In addition to IDD, ASD and ADHD phenotypes are often observed in Smith Magenis syndrome, with *RAI1* carriers affected even more frequently despite a higher cognitive level. This systematic review highlights the need to systematically screen people with Smith Magenis syndrome for attention deficit hyperactivity disorder and autism spectrum disorder in addition to cognitive functions. Smith Magenis syndrome is a relevant model to further study physiopathology of neurodevelopmental disorder.

**Trial registration:**

(PROSPERO no.: CRD42024512675).

**Supplementary Information:**

The online version contains supplementary material available at 10.1186/s11689-026-09707-y.

## Background

Neurodevelopmental disorders, as described in the Diagnostic and Statistical Manual of Mental Disorders, Fifth Edition (DSM-5), include Autism Spectrum Disorder (ASD), Attention-Deficit Hyperactivity Disorder (ADHD), Specific learning disability and Intellectual Developmental Disorder (IDD) [1]. Smith-Magenis Syndrome (SMS) is a rare genetic disorder either due to a deletion in 17p11.2 locus [2] or to a pathogenic variant in *RAI1* gene [3], causing a unique melatonin circadian secretion disorder [4, 5]. It is associated with a higher risk of neurodevelopmental disorder, with most subjects scoring in the moderate IDD level [6]. Since the first description of SMS, it has been associated with maladaptive behaviour [7]. Later research has helped describe the wide range of maladaptive behaviour: aggression, temper tantrums and outbursts, hyperactivity, impulsivity, attention deficits and attention-seeking behaviours with attachment to a preferential adult, self-hugging, polyembolokoilamania, head banging, wrist biting, skin picking, onychotillomania, hand licking and page flipping are often observed early in development [6, 8–15]. Interestingly, positive behaviour seems to increase with age while negative behaviour decreases [16]. A recent scoping review focusing on psychiatric and neurological manifestations of SMS in adults reported ASD- and ADHD-related features in approximately 40% of the 19 cases-studies cited, with only two individuals having a formal diagnosis of ASD and two of ADHD [17]. Except for higher Full Scale Intelligence Quotient (FSIQ) and more internalizing behaviours reported for *RAI1* mutations carriers compared to deletion 17p11.2 [18], no neurodevelopmental genotype-phenotype correlation has been described. Still, whereas behavioural and neurodevelopmental issues are known, prevalence of psychiatric and neurodevelopmental conditions are lacking. We performed a systematic review to assess the prevalence of neurodevelopmental disorders (autism spectrum disorder, attention deficit hyperactivity disorder, intellectual developmental disorder, specific learning disabilities) in SMS as a main outcome. We described the methods used to identify a neurodevelopmental disorder in this population and assessed a phenotype-genotype correlation as secondary outcomes.

## Methods

This review complied with the Preferred Reporting Items for Systematic Reviews and Meta-Analyses (PRISMA) guidelines [19], and the protocol was pre-registered with PROSPERO (CRD42024512675).

### Search strategy

Main electronic databases (PubMed [including MEDLINE], Cochrane Library [Wiley], Web of Science [Clarivate Analytics] and Embase [Elsevier]) were searched by our specialized librarian on January, 31st 2024 (see appendix 1 for queries).

### Eligibility criteria

We considered original articles published in peer-reviewed journal. We selected studies with data about prevalence of neurodevelopmental disorders in people with SMS, assessed by an experienced team and/or relevant psychometric tools. Case reports and reviews were excluded. Quality was assessed with an adapted version of the quality framework proposed by Richards et al. [20] and Thomas et al. [21], based on sample identification, confirmation of syndrome, assessment of neurodevelopmental disorders and data coverage (see appendix 4).

### Selection process

Two researchers (PB, MNB) independently reviewed all titles and abstracts, and independently screened titles and abstracts of all articles retrieved. In case of disagreement, consensus on which articles to screen full-text was reached by discussion. If necessary, a third researcher (CD) was consulted to reach the final decision. PB and MNB then independently screened full-text articles for inclusion. Again, in case of disagreement, consensus was reached on inclusion or exclusion by discussion and if necessary, CD was consulted.

### Data extraction

We designed a data extraction form, which two review authors (PB and MNB) used to extract data from eligible studies. Extracted data were compared, with any discrepancies being resolved through discussion. When information regarding any of the above was unclear, we contacted authors of the reports to provide further details.

We collected data on:

- Author(s) and year of publication
- Type of study
- Age and gender of population
- Genotype
- Prevalence of diagnosis and traits of neurodevelopmental disorders
- Age at assessment
- Tools used to assess the neurodevelopmental phenotype
- Type of neurodevelopmental disorder
- Comorbid organic or psychiatric disorders
- Comorbid genetic variation

### Risk of bias assessment

Amendment to PROSPERO protocol: We initially stated “The Munn et al.’s tool for use in systematic review addressing questions of prevalence” [22] will be used to assess for quality of studies. Because it was more relevant in the context of rare diseases; we decided, instead, to use an adapted version of the quality framework from Richards et al. [20] and Thomas et al. [21] (see appendix 4). Four specific domains were assessed: (1) sample identification; (2) confirmation of syndrome; (3) assessment of neurodevelopmental disorders; (4) data exhaustiveness. The first author applied the tool to each included study and recorded supporting information and justifications for quality appraisal for each domain (ranging from 1: poor; to 4: excellent). An overall score was awarded based on the four specific domains scores (Table 5).

## Results

### Systematic literature research

**Fig. 1:**
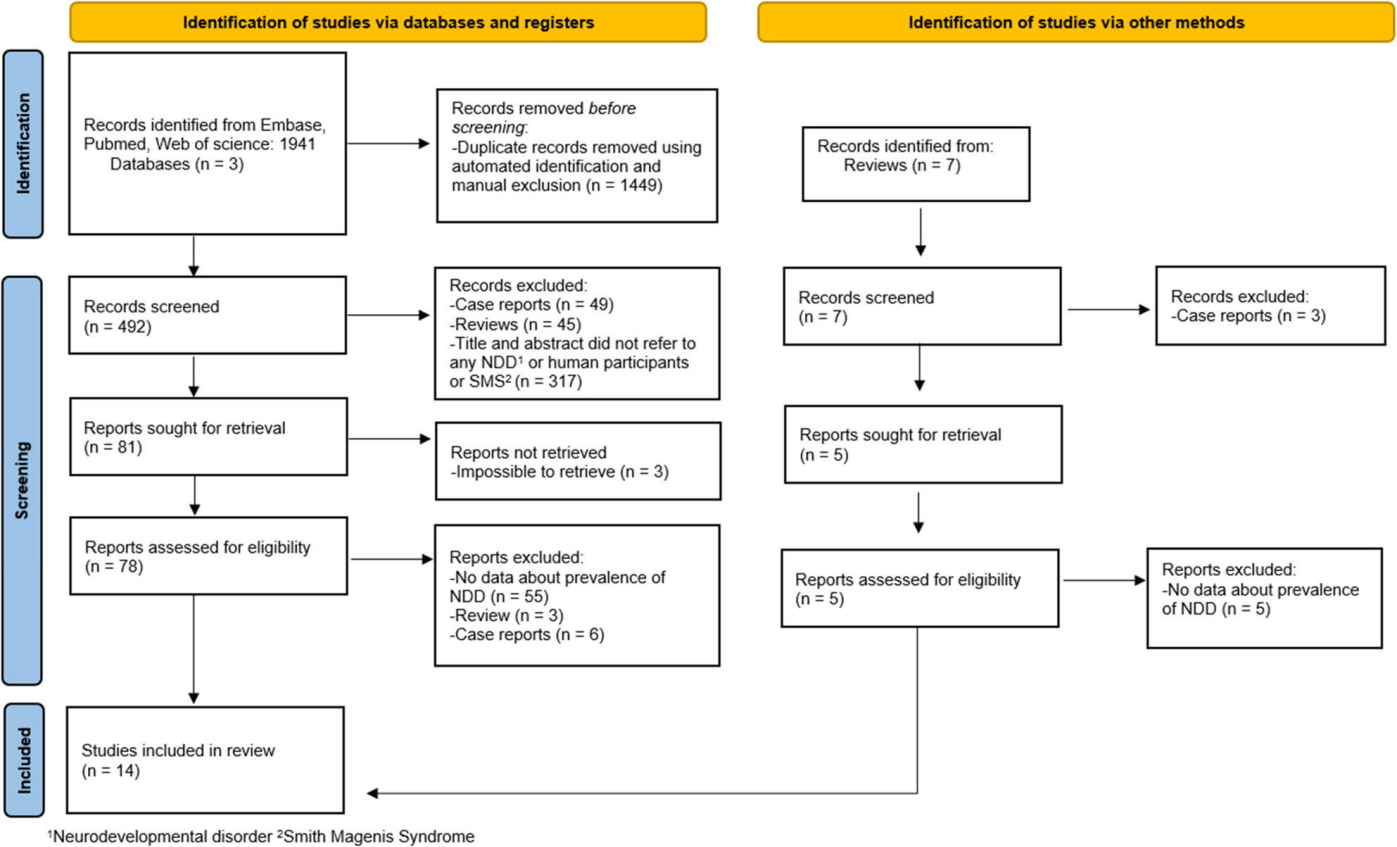
PRISMA 2020 flow diagram presenting papers included and excluded at each stage of the selection process

The search yielded 1941 articles, while 7 citations were identified from reviews. 14 studies were included in this review after selection process (Fig. 1).

### Studies and population characteristics

In total, 451 patients presenting SMS were assessed for neurodevelopmental disorders, from 9 different countries: The Netherlands, USA, UK, France, Brazil, Norway, Sweden, Portugal and Spain (Table 1). Mean age was 13.9 years old (ranging from 1.5 to 51 years old); 51% were male and 32 patients had *RAI1* mutation.

**Table 1:** Description of studies and population characteristics

Study	Age (y) mean/median (range)	Country (cohort)	Sample size	Male (%)	Genotype *n* (%)	
					Del17p11.2	RAI1
Linders & al, 2023 [18]	-/- (2 to 45)	The Netherlands	66	48%	47 (71)	19 (29)
Martin & al, 2006 [23]	5.7/- (2 to12)	USA (Maryland, NIH 01-HG-0109)	19	47%	19 (100)	
Oliver & al, 2011 [24]	15.45/- (4–38)	UK (Birmingham, Smith Magenis Foundation)	42	40.5%		
Nag & al, 2018 [25]	18.5/- (5.1–50.5)	Norway & Sweden	28	46%	25 (89)	3 (11)
Osório & al, 2012 [26]	14/- (7–29)	Portugal & Spain	15	53%	14 (93)	1 (7)
Osório & al, 2013 [27]	13.8/- (2.8–25)	Portugal & Spain	19	53%	19 (100)	
Osório & al, 2015 [28]	13.18/- (8–19)	Portugal & Spain	11	55%	11 (100)	
Rive Le Gouard & al, 2020 [29]	11.4/10.4 (4–36)	France	47	40%	47 (100)	
Sloneem & al, 2011 [11]	15.09/- (6–39)	UK (Birmingham, Smith Magenis Foundation)	32	43.80%		
Vieira & al, 2012 [30]	22.8/- (-)	Brazil	7	57%	7 (100)	
Laje & al, 2010 [31]	14.4/- (4.2–49.9)	USA (Maryland, NIH 01-HG-0109)	26	42%	26 (100)	
Alaimo & al, 2015 [12]	13.5/- (1,5–51)	USA (PRISMS)	99	41%	90 (90)	9 (10)
Dykens & al, 1998 [10]	9.57/- (4–20)	USA (PRISMS)	35	43%	35 (100)	
Boddaert & al, 2004 [39]	13.3/11.5 (11.5–13.5)	France	5	100%	5 (100)	
Total	13.9/- (1.5–51)		451	51%	419 (86)	32 (14)

### Prevalence of autism spectrum disorder characteristics

We extracted data from 6 studies assessing ASD characteristics from 6 different cohorts for a total of 220 patients (Table 2). 168 had a deletion and 10 had a reported *RAI1* mutation, one study did not specify genetic status. Reported prevalence of ASD characteristics ranged from 14% to 95%, with a minimum of 52% when selecting studies using standardized tools. Mean prevalence is 58% for total sample, with 43% and 80% for deletion and *RAI1* samples respectively. Methods of assessment were heterogeneous: one study used the Child Behaviour CheckList (CBCL), one the Autism Screening Questionnaire (score above 15 indicates ASD and a score above 22 is considered as Autism), three studies reported using the Social Communication Questionnaire (SCQ, Lifetime score ≥ 15 indicates ASD) and one study was based on a parental form. Additionally, severity of ASD was also explored in 3 studies, one using the current score of the SCQ with 35% of patients scoring above pathological cut-off, and 2 other studies using the Social Responsiveness Scale (severity of social impairment). In these studies, 90% to 96% of patients showed impairment. (appendices 2 and 3).

**Table 2:** Prevalence and assessment of autism spectrum disorder characteristics

Study	Country	Sample (*n*)	Method of assessment	Autism spectrum disorder % (*n*)		
				Total sample	Del17p11.2	RAI1
Linders & al, 2023 [18]	The Netherlands	66	CBCL^a^-Autistic Traits Profile	75% (18/24)	71% (10/14)	80% (8/10)
Oliver & al, 2011 [24]	UK	42	ASQ^b^	95% (40/42) *ASD*^*c*^: *65% (26/40) Autism: 35% (14/40)*		
Nag & al, 2018 [25]	Norway & Sweden	28	SCQ^d^-Lifetime	52% (14/27)	52% (14/27)	
Osório & al, 2015 [28]	Portugal & Spain	11	SCQ-Lifetime	73% (8/11)	73% (8/11)	
Rive Le Gouard & al, 2020 [29]	France	47	Parents report	14% (6/43)	14% (6/43)	
Laje & al, 2010 [31]	USA	26	SCQ-Lifetime	54% (14/26)	54% (14/26)	
Total		220		58% (100/173)	43% (52/121)	80% (8/10)

^a^*CBCL* Child Behaviour CheckList, ^b^*ASQ* Autism Screening Questionnaire, ^c^*ASD* Autism Spectrum Disorder, ^d^*SCQ* Social Communication Questionnaire

### Prevalence of attention deficit hyperactivity disorder & traits

6 studies assessed ADHD and related traits, for a total of 174 patients from 7 different countries (Table 3). 122 had a deletion and 10 had a *RAI1* mutation. Only one study assessed ADHD using standardized clinical criteria (DSM-IV) and reported 100% prevalence. Other studies assessed ADHD symptoms (impulsivity, hyperactivity, attention deficit and lability); using the CBCL, structured interviews, The Activity Questionnaire or clinical reports. Pathological scores of impulsivity ranged from 49% to 86% and hyperactivity varied between 11.3% and 100%. Prevalence of attention deficit was reported with 93% and 94.7% for deletion carriers and 100% for *RAI1* mutation carriers.

**Table 3:** Prevalence and assessment of attention deficit hyperactivity disorder diagnosis & traits

Study	Country	Sample (*n*)	Method of assessment	Attention deficit hyperactivity disorder % (*n*)		
				Total sample	Del17p11.2	RAI1
Linders & al, 2023 [18]	The Netherlands	66	CBCL^a^ attention problem scale	96% (23/24)	93% (13/14)	100% (10/10)
Oliver & al 2011 [24]	UK	42	TAQ^b^	Impulsivity: 49% *children: 40% adults: 58.3%* Hyperactivity: 11.3% *children: 6.9% adults: 16.7%*		
Osório & al, 2013 [27]	Portugal & Spain	19	Structured interviews with close relatives	Attention deficit: 94.7% (18/19) Hyperactivity: 68.4% (13/19)	Attention deficit: 94.7% (18/19) Hyperactivity: 68.4% (13/19)	
Vieira & al, 2012 [30]	Brazil	7	Clinical report	Hyperactivity: 100% (4/4)	Hyperactivity: 100% (4/4)	
Dykens & al, 1998 [10]	USA	35	CBCL	Hyperactivity: 94–100% Lability: 89% (31/35) Impulsivity: 86% (30/35)	Hyperactivity: 94–100% Lability: 89% (31/35) Impulsivity: 86% (30/35)	
Boddaert & al, 2004 [39]	France	5	DSM-IV^c^	100% (5/5)	100% (5/5)	
Total		174		Attention deficit: 96% (46/48) Impulsivity: 78% (-) Hyperactivity: 75%-76% (-) Lability: 89% (31/35)	Attention deficit: 95% (36/38) Hyperactivity: 91–92% (-) Impulsivity: 87.5% (35/40) Lability: 89% (31/35)	100% (10/10)

^a^*CBCL* Child Behaviour CheckList, ^b^*TAQ* The Activity Questionnaire, ^c^*DSM-IV* Diagnostic and Statistical Manual of Mental Disorders

### Prevalence of intellectual developmental disorder and specific learning disability

9 studies reported IDD prevalence, ranging from 67% to 100% (Table 4). For deletion carriers, pooled results showed a 93% prevalence of IDD, mostly moderate (43%), while *RAI1* mutation carriers had 80% IDD, mostly mild (69%). Only one study reported 9% (9/43) of specific learning disability (appendix 2). No information about specific learning disabilities was otherwise specified. 10 different tools were used to assess cognitive function, depending on age and cognitive impairment.

**Table 4:** Prevalence and assessment of IDD & specific learning disability

Study	Country	Sample (*n*)	Method of assessment	Intellectual disability disorder % (*n*)		
				Total sample	Del17p11.2	RAI1
Linders & al, 2023 [18]	The Netherlands	66	based on lifetime clinical records, school reports, FSIQ^a^ scores, and according to the DSM-5^b^	92% (49/53)	97% (33/34) *Mild IDD: 34% (11/33) Moderate IDD: 48% (16/33)* *Severe IDD: 18% (6/33)*	84% (16/19) *Mild IDD: 69% (11/16) Moderate IDD: 19% (3/16) Severe IDD: 12% (2/16)*
Martin & al, 2006 [23]	USA (Maryland, NIH 01-HG-0109)	19	BSID-II^c^, SBIS-IV^d^, VABS^e^	67% (12/18)	67% (12/18) *Mild IDD: 50% (6/12) Moderate IDD: 50% (6/12)*	
Nag & al, 2018 [25]	Norway & Sweden	28		75% (21/28) *Mild IDD: 24% (5/21) Moderate IDD: 71% (15/21) Severe IDD: 5% (1/21)*		
Osório & al, 2012 [26]	Portugal & Spain	15	WISC-IV^f^, WAIS-III^g^	93% (14/15)	100% (14/14) *Mild IDD: 14% (2/14) Moderate IDD: 86% (12/14)*	0% (0/1)
Rive Le Gouard & al, 2020 [29]	France	47	WPPSI^h^, WISC-IV, WAIS, EDEI-R^i^, PEP-3^j^, Brunet-Lézine Scale	91% (39/43) *Mild IDD: 23% (9/39) Moderate IDD: 67% (26/39) Severe IDD: 3% (1/39) Profound IDD: 7% (3/39)*	91% (39/43) *Mild IDD: 23% (9/39) Moderate IDD: 67% (26/39) Severe IDD: 3% (1/39) Profound IDD: 7% (3/39)*	
Sloneem & al, 2011 [11]	UK	32	WISC-III, WAIS-III, VABS	100% (32/32) *Mild IDD: 28*,*1% (9/32) Moderate IDD: 28*,*1% (9/32) Severe/Profound IDD: 43*,*8% (14/32)*		
Vieira & al, 2012 [30]	Brazil	7	Clinical report	100% (7/7)	100% (7/7)	
Dykens & al, 1998 [10]	USA (PRISMS)	35	VABS	100% (35/35)	100% (35/35)	
Boddaert & al, 2004 [39]	France	5	WISC-R	100% (5/5) *Mild IDD 40% (2/5) Moderate IDD 60% (3/5)*	100% (5/5) *Mild IDD 40% (2/5) Moderate IDD 60% (3/5)*	
Total		254		86% (202/236) *Mild IDD 13% (25/202)* *Moderate IDD 26% (53/202)* *Severe/Profound IDD 9% (19/202)* *Unspecified 52% (105/202)*	93% (145/156) *Mild IDD 23% (33/145)* *Moderate IDD 43% (63/145)* *Severe IDD 5% (7/145)* *Profound IDD 2% (3/145)* *Unspecified 27% (39/145)*	80% (16/20) *Mild IDD: 69% (11/16) Moderate IDD: 19% (3/16) Severe IDD: 12% (2/16)*

^a^*FSIQ* Full-Scale Intelligence Quotient, ^b^*DSM*-5 Diagnostic and Statistical Manual of Mental Disorders, Fifth edition, ^c^*BSID-II* Bayley Scale of Infant Development, Second edition, ^d^*SBIC-IV* Stanford Binet Intelligence Scale, Fourth edition, ^e^*VABS* Vineland Adaptative Behaviour Scale ^f^*WISC-IV* Wechsler Intelligence Scales for Children, Fourth edition, ^g^*WAIS*- *III* Wechsler Adult Intelligence Scales-Third edition, ^h^Wechsler Preschool and Primary Scale of Intelligence ^i^*EDEI-R* the Differential Scales of Intellectual Efficiency-Revised edition, ^j^*PEP-3* Psychoeducational Profile-third edition

### Quality assessment

Most studies stated adequate sample identification; good confirmation of syndrome and excellent data coverage (Table 5). Neurodevelopmental disorder assessment varied from poor (4 studies) to excellent (1 study), with a large majority of adequate evaluation based on standardized informant report measures or DSM criteria (9 studies).

**Table 5:** Quality assessment of studies

Study	Sample identification	Confirmation of Syndrome	Neurodevelopmental disorder assessment	Data coverage	Overall
Linders & al, 2023 [18]	Adequate	Good	ASD&ADHD: adequate	Adequate	Adequate
			IDD: excellent		
Martin & al, 2006 [23]	Adequate	Excellent	Adequate	Excellent	Good
Oliver & al 2011 [24]	Adequate	Poor	Adequate	Excellent	Adequate
Nag & al, 2018 [25]	Good	Good	ASD: adequate	Excellent	Good
			IDD: poor		
Osório & al, 2012 [26]	Adequate	Good	Good	Excellent	Good
Osório & al, 2013 [27]	Adequate	Good	Poor	Excellent	Adequate
Osório & al, 2015 [28]	Adequate	Good	Adequate	Excellent	Good
Rive Le Gouard & al, 2020 [29]	Excellent	Good	ASD: poor	Excellent	Good
			IDD: good		
Sloneem & al, 2011 [11]	Adequate	Poor	Good	Excellent	Adequate
Vieira & al, 2012 [30]	Good	Excellent	Poor	Excellent	Good
Laje & al, 2010 [31]	Adequate	Good	Adequate	Excellent	Good
Alaimo & al, 2015 [12]	Adequate	Good	Adequate	Excellent	Good
Dykens & al, 1998 [10]	Adequate	Good	Adequate	Excellent	Good
Boddaert & al, 2004 [39]	Adequate	Excellent	ADHD: Adequate	Excellent	Good
			IDD: good		

## Discussion

This is the first systematic review describing prevalence of neurodevelopmental disorders in Smith-Magenis Syndrome. Strengths of our study are the use of key terms specific to neurodevelopmental conditions and the systematic approach following PRISMA guidelines.

Our results show strikingly high proportions of ASD and ADHD characteristics alongside IDD. ASD characteristics are described in 58% of cases overall, reaching 80% in the *RAI1* sample. Attention deficit, impulsivity and hyperactivity are respectively reported in 96%, 78% and 75% of cases. Interestingly, ADHD characteristics rate is even higher in the *RAI1* cohort with 100% prevalence. These results are consistent with previous findings: Edelman et al. reported 74.2% of hyperactivity in their cohort, with higher prevalence of hyperactivity for *RAI1* (100%, 7/7) compared to deletions (44.4%, 49/66) [32].

These results highlight the need to systematically screen for ADHD and ASD in SMS population, in addition to assess for cognitive performance. Indeed, to make a clear and precise diagnosis is essential for the announcement of medical diagnosis and for the information given to families, as well as for the organization of relevant support. It is critical to manage ADHD and ASD special needs, especially when associated with behavioural issues typically described in SMS. Sensory processing difficulties have previously been reported in a cohort of 34 children with SMS across all Sensory Profile categories, with a trend toward extreme sensory processing behaviours in older females and an association with impaired behavioural regulation [33]. Following the high prevalence of ADHD characteristics, potential positive impact of methylphenidate in SMS could be assessed in further research.

Cognitive profile in SMS has been widely described, with most cases showing moderate IDD and a more severe degree of IDD observed in deletion 17p11.2 carriers in comparison to *RAI1* pathogenic variant carriers. The results of this review are consistent with these findings. However, our study emphasizes the heterogeneity of cognitive assessment with lack of standardization. Indeed, one study shows that deletion 17p11.2 is not systematically associated with IDD, with one case of specific learning disability. This is an important issue in rare disease landscape: despite the expected phenotype, we highlight the importance to systematically evaluate with standardized tools the cognitive profile.

Although attention deficit, impulsivity and hyperactivity are all reported in very high proportions, formal diagnosis of ADHD is rarely available. The lack of formal diagnosis may be explained by the difficulty of assessing ADHD in people with IDD since attention and behaviour can be affected by cognitive functioning [34] and DSM-5 criteria shared between the two neurodevelopmental conditions [35]. Thus, it is essential to evaluate attention, hyperactivity and impulsivity levels in people with IDD by taking into account their developmental age more so than the chronological age. Moreover, international guidelines for assessment of comorbid ADHD state that the use of valid tools such as DIVA-ID [36] or multidisciplinary approaches and clinical judgement are relevant [35, 37]. Another issue that may influence ADHD prevalence is the presence of comorbid sleep disorders. Nycthemeral inversion is well described in SMS and is associated with sleep disturbances. Included studies did not report additional data about sleep quality, warranting caution when interpreting the specificity of this high prevalence. Still, sleep and ADHD share a complex bidirectional relationship, and treating ADHD can improve sleep quality [38] : having a formal diagnosis of ADHD can help caregivers and patients receive the appropriate non-pharmacological and pharmacological care.

The careful assessment of neurodevelopmental disorder phenotypes in SMS can help further understand the physiopathology underlying these conditions. Interestingly, bilateral decrease of grey matter concentration in insulo-lenticular regions in individuals with SMS compared to controls has been described [39] while involvement of these areas has been suggested in patients with ADHD [40]. This further suggests that SMS may be a relevant model for further research on syndromic ADHD.

Regarding autism and SMS, it is important to emphasize that distinguishing between behavioural characteristics resulting from ASD or IDD is a complex clinical endeavour. This challenge is further heightened in the context of rare diseases such as SMS: are these features core ASD symptoms, intrinsic traits of SMS, or consequences of developmental delay?

Previous studies have proposed clinical guidance to improve the specificity and sensitivity of assessments to distinguish IDD and ASD. First, careful evaluation of the cognitive profile is essential, with caution when interpreting potentially artificially low performance in individuals with ASD or ADHD due to interfering symptomatology. Second, social skills development should be considered in relation to developmental age: can social impairments be better explained by overall developmental delay? Finally, the developmental trajectory should be assessed: were social impairments and repetitive behaviours present during infancy? How did these features evolve with overall development? Did the gap between social and other developmental skills widen over time [41, 42]? 

The lack of longitudinal studies, particularly those including adults, limits the ability to draw firm conclusions regarding the specificity and extent of the high prevalence rates observed in this systematic review. However, the even higher prevalence of ADHD and ASD characteristics reported in *RAI1* carriers—despite a relatively preserved cognitive profile—supports the hypothesis that these features may represent core symptoms of SMS rather than being solely attributable to IDD.

In addition to sensitivity and specificity issues, it is important to note that the intrinsic ASD phenotype in SMS may be considered atypical : reversed gender ratio has been described, with altered social communication despite a friendly phenotype [25]. With a mean of 58% of patients displaying ASD phenotype in our results, further understanding of the specific features overlapping with ASD profiles in SMS is needed. Despite the high prevalence of autism characteristics, only screening tools were used. As stated by Korteling et al., the Autistic Diagnostic Interview (ADI) and the Autistic Diagnostic Observation Scale (ADOS) have indeed not been validated for SMS population [17]. Some research suggested that SCQ-L is a relevant tool for ASD assessment in genetic syndromes, with high correlations to ADOS and ADI-R(42). Despite the ambiguity regarding specificity of ASD profile overlapping with specific genetic traits, having a formal diagnosis of ASD may help families and children in receiving the most appropriate care [43].

Although no genotype-phenotype correlations can be ascertained due to small samples, it is interesting to note that despite a higher level of cognitive function, individuals with *RAI1* mutations tend to display higher proportions of ASD and ADHD.

Limits are the low number of studies included and relatively low quality of assessment of neurodevelopmental disorders, with high heterogeneity and lack of standardization.

Indeed, the paucity of studies did not allow for the conduction of a proper meta-analysis or quality-weighted analysis. However, the overall study quality was adequate, and discrepancies across the four criteria should be considered in the context of a rare disease: sample identification by randomization or screening within the general population for example, would be an almost impossible study design due to the low expected prevalence of SMS.

Confirmation of the syndrome depends on the diagnostic techniques available at the time of each study, and SMS is not considered a clinically challenging diagnosis for trained geneticists, thereby limiting the risk of misdiagnosis. Two of the included studies, Sloneem et al. [11] and Oliver et al. [24] exhibit poor ‘confirmation of syndrome’ criteria quality. Notably, Oliver et al. [24] reported the lowest prevalence of ADHD characteristics and the highest prevalence of ASD characteristics, suggesting that a quality-weighted analysis might have yielded even higher ADHD rates but more moderate ASD phenotype results.

The precise assessment of NDDs generally requires the use of standardized questionnaires and assessment tools specifically designed for the population of interest. To our knowledge, no specific screening tool has yet been developed to assess NDDs in SMS. The assessments reported in this systematic review rely on general population-based psychometric scales, unconfirmed diagnoses based on parental or clinical reports, or clinical judgement. This may substantially affect the reliability of estimates. Interestingly, the included study with poorer ASD assessment quality, Rive Le Gouard et al. [29] also reported the lowest prevalence rate, suggesting that a quality-weighted analysis might have identified higher rates of ASD characteristics. Included studies with poorer ADHD and IDD assessment quality, Nag et al. [25], Vieira et al. [30], and Osório et al. [27] did not differ from expected results.

Data coverage was excellent, limiting the risk of selective reporting and supporting overall reliability.

Further research is needed to better understand the extent and specificity of NDDs phenotypes and relevant clinical care in SMS.

## Conclusion

High proportions of ADHD and ASD characteristics are described in SMS. Despite a higher cognitive functioning, individuals with *RAI1* mutations tend to display ASD and ADHD phenotypes even more frequently. Systematic assessment of ASD and ADHD diagnosis and symptoms is relevant for people with SMS, in addition to careful assessment of cognitive function. Giving information about higher risk of occurrence of these symptoms is critical for families and children’s care. Further research is needed to assess whether diagnostic tools and management guidelines for ASD and ADHD are adequate in context of SMS.

## Supplementary Information

Supplementary Material 1.

Supplementary Material 2.

## Data Availability

No datasets were generated or analysed during the current study.

## Abbreviations

ADI: Autistic Diagnostic Interview
ADHD: Attention-Deficit Hyperactivity Disorder
ADOS: Autistic Diagnostic Observation Scale
ASD: Autism Spectrum Disorder
CBCL: Child Behaviour CheckList
DSM-5: Diagnostic and Statistical Manual of Mental Disorders, Fifth Edition
FSIQ: Full Scale Intelligence Quotient
IDD: Intellectual Developmental Disorder
SCQ: Social Communication Questionnaire
SMS: Smith-Magenis syndrome
