# Supplementary material for: The prevalence of neurodevelopmental disorders in Smith-Magenis Syndrome: a PRISMA compliant systematic review
Source: J Neurodev Disord. 2026 Jun 1;18:47. doi: 10.1186/s11689-026-09707-y (PMC13435750; doi:10.1186/s11689-026-09707-y)
Supplement: Supplementary file 1 [file 11689_2026_9707_MOESM1_ESM.docx]

**Supplementary Material**

**Appendix 1.**

Research queries were completed on January 31^st^, 2024:

1 – **Autism** ((("Child Development Disorders, Pervasive"[MH] OR autis*[TI] OR "pervasive developmental disorder*"[TI] OR "pdd-nos"[TI] OR pdd[TI] OR asperger[TI] OR aspergers[TI] OR "asperger's"[TI] OR "Childhood disintegrative disorder"[TI] OR "Pervasive development disorder"[TI] OR ASDs [TI] OR ASD[TI] OR "asd's"[TI])) AND ("Smith-Magenis Syndrome"[MH] OR "Smith-Magenis"[TI] OR "17p11.2"[TI] OR "RAI1"[TI])) = **22 results**

2 – **ADHD** ((("Attention Deficit Disorder with Hyperactivity"[MH] OR "Hyperkinesis"[MH] OR hyperactiv* [TI] OR ADHD[TI] OR "adhd's"[TI] OR "AD/HD"[TI] OR "ADH/D"[TI] OR "Attention Deficit"[TI] OR hyperkine*[TI] OR ADHS[TI] OR ADD[TI] OR ADDH[TI] OR inattenti*[TI])) AND ("Smith Magenis Syndrome"[MH] OR "Smith-Magenis"[TI] OR "17p11.2"[TI] OR "RAI1"[TI])) = **9 results**

3 – **IDD** Two strategies proposed: The first combining the MESH terms and the words included in the title. The second combines the MAJOR MESH terms for IDD with the terms included in the title ((("Intellectual Disability"[MH] OR "Intellectual Disab*"[TI] OR "Intellectually Disab*"[TI] OR "Intellectual deficien*"[TI] OR "Intellectually deficien*"[TI] OR "Intellectual retard*"[TI] OR "Intellectually retard*"[TI] OR "mental disab*"[TI] OR "mentally disab*"[TI] OR "mental retard*"[TI] OR "mentally retard*" OR "mental ill*"[TI] OR "mentally ill*"[TI] OR "mental deficien*"[TI] OR "mentally deficien*"[TI] OR "Intellectual and Developmental disab*"[TI] OR "Developmental and Intellectual disab*"[TI])) AND ("Smith-Magenis Syndrome"[MH] OR "Smith-Magenis"[TI] OR "17p11.2"[TI] OR "RAI1"[TI])) **= 142 results** ((("Intellectual Disability"[MAJR] OR "Intellectual Disab*"[TI] OR "Intellectually Disab*"[TI] OR "Intellectual deficien*"[TI] OR "Intellectually deficien*"[TI] OR "Intellectual retard*"[TI] OR "Intellectually retard*"[TI] OR "mental disab*"[TI] OR "mentally disab*"[TI] OR "mental retard*"[TI] OR "mentally retard*" OR "mental ill*"[TI] OR "mentally ill*"[TI] OR "mental deficien*"[TI] OR "mentally deficien*"[TI] OR "Intellectual and Developmental disab*"[TI] OR "Developmental and Intellectual disab*"[TI])) AND ("Smith- Magenis Syndrome"[MAJR] OR "Smith-Magenis"[TI] OR "17p11.2"[TI] OR "RAI1"[TI])) **= 94 results**

4 – **NDD** Two strategies proposed: The first combining the MESH terms and the words included in the title. The second combines the MESH term “Neurodevelopmental Disorders” without explosion of terms and the words included in the title. (("Neurodevelopmental Disorders"[MH] OR "Neurodevelopmental Disorder*"[TI] OR neurodevelopment*[TI] OR "neuro-development*"[TI] OR NDD[TI]) AND ("Smith-Magenis Syndrome"[MH] OR "Smith-Magenis"[TI] OR "17p11.2"[TI] OR "RAI1"[TI])) = **169 results** (("Neurodevelopmental Disorders"[MH:noexp] OR "Neurodevelopmental Disorder*"[TI] OR neurodevelopment*[TI] OR "neuro-development*"[TI] OR NDD[TI]) AND ("Smith-Magenis Syndrome"[MH] OR "Smith-Magenis"[TI] OR "17p11.2"[TI] OR "RAI1"[TI])) **= 13 results**

5 – **Specific learning disabilities** ("Learning Disabilities"[MH] OR "Learning Disabilities"[TI] OR "Learning Disabilit*"[TI] OR "learning disorder*"[TI] OR "learning impair*"[TI] OR Dyslexi*[TI] OR Dysgraphi*[TI] OR Dyscalculi*[TI]) AND ("Smith-Magenis Syndrome"[MH] OR "Smith-Magenis"[TI] OR "17p11.2"[TI] OR "RAI1"[TI]) **= 4 results**

6 – **Impulsivity** ("Impulsive Behavior"[MH] OR impulsi*[TI]) AND ("Smith-Magenis Syndrome"[MH] OR "Smith- Magenis"[TI] OR "17p11.2"[TI] OR "RAI1"[TI]) **= 0 result**

7 – **Challenging behavior** ("Challenging behavior"[TI] OR "behavior disorder"[TI] OR "behaviour disorder"[TI] OR "Child Behavior Disorders"[MH]) AND ("Smith-Magenis Syndrome"[MH] OR "Smith-Magenis"[TI] OR "17p11.2"[TI] OR "RAI1"[TI]) = **18 results**

8 – **Aggressivity** ("aggressive behavi*"[TI] OR "agressive behavi*"[TI] OR aggression[MH]) AND ("Smith Magenis Syndrome"[MH] OR "Smith-Magenis"[TI] OR "17p11.2"[TI] OR "RAI1"[TI]) **= 9 results**

9 – **Social interactions** ("Social Interaction"[MH] OR "Social Interaction*"[TI]) AND ("Smith-Magenis Syndrome"[MH] OR "Smith-Magenis"[TI] OR "17p11.2"[TI] OR "RAI1"[TI]) 3 = 1 Résultat :

10 – **Repetitive behavior** ("Stereotyped Behavior"[MH] OR "Stereotyp*"[TI] OR "Repetitive behavi*"[TI]) AND ("Smith- Magenis Syndrome"[MH] OR "Smith-Magenis"[TI] OR "17p11.2"[TI] OR "RAI1"[TI]) = **4 results**

11 – **Sexual behavior** ("Inappropriate sexual behavior"[TI] OR "sexual behavior"[MH] OR "sexual behavi*[TI]) AND ("Smith-Magenis Syndrome"[MH] OR "Smith-Magenis"[TI] OR "17p11.2"[TI] OR "RAI1"[TI]) = **0 result**

**Appendix 2.**

Table 1 displays all extracted data, including age of assessment, comorbid conditions and additional behavioral informations.

***Insert excel here***

**Appendix 3.**

We report here detailed scoring methods regarding autism:

- CBCL scales from Linders&al were correlated to DSM-IV disorders. We extracted raw CBCL data and extrapolated ADHD and ASD traits proportions using cut-off scores described in previous studies. Social Problems score + Thought Problems score + Withdraw/Depressed score ≥195 indicates ASD

- Autism Screening Questionnaire: score above 15 indicates ASD and above 22 is considered Autism

- Social Communication Questionnaire: Lifetime score ≥15 indicates ASD

**Appendix 4.** Quality appraisal

The risk of methodological bias between the individual studies included was assessed with an adapted version of the quality framework from Richards et al. and Thomas et al. We simplified the framework of the original work by merging the psychological/behavioural and cognitive assessments into a unique category named “neurodevelopmental disorders assessment”. We added a fourth domain assessing data exhaustivity.

The adapted quality criteria are presented in Table 2. We used the original quality framework criteria for sample identification, confirmation of syndrome and neurodevelopmental disorders assessment domains. In regard to data exhaustivity, ratings were proportional to sample coverage.

Table 2 Risk of bias framework

|  | 1 – Poor quality, High risk of bias | 2-Adequate quality, some concerns about risk of bias | 3-Good quality, some concerns about risk of bias | 4-Excellent quality, low risk of bias |
| --- | --- | --- | --- | --- |
| Sample identification | Not specified/reported | Single, restricted or non-random sample  (e.g. from a specialist clinic or previous  research study) | Multiple restricted or non-random sample  (e.g. multiregional specialist clinics) | Random or total population sample |
| Confirmation of syndrome | Not reported or reported without reference  to the criteria use | Specific clinical criteria reported without genetic assessment | Specific clinical criteria reported with genetic assessment reported  retrospectively | Specific clinical criteria reported  alongside prospective genetic  assessment |
| NDD^1^ assessment | Descriptions of NDD based on  non-standardised informant report or  review of clinical information | Standardised informant report measure  (e.g. VABS^2^  ADI-R^3^ or Conners rating scales) or clinical judgement  based on DSM^4^  criteria) | Standardised behavioural or observational  assessment (e.g., neuropsychiatric  evaluation, ADOS^5^, Weschler intelligence scales) | Consensus drawn from multiple assessments,  including one or more standardised  behavioural or observational  assessment |
| Data coverage | < 25 % sample coverage | 25% < x < 50% sample coverage | 50 % < x < 75 % sample coverage | > 75% sample coverage |

^1^ Neurodevelopmental disorder

^2^ Vineland Adaptative Behaviour Scale

^3^ Autism Diagnostic Interview-Revised

^4^ Diagnostic and Statistical Manual of Mental Disorders

^5^ Autism Diagnostic Observation Schedule
